# The impact of alcohol taxation increase on all-cause mortality inequalities in Lithuania: an interrupted time series analysis

**DOI:** 10.1186/s12916-022-02721-6

**Published:** 2023-01-16

**Authors:** Jakob Manthey, Domantas Jasilionis, Huan Jiang, Olga Meščeriakova, Janina Petkevičienė, Ričardas Radišauskas, Mindaugas Štelemėkas, Jürgen Rehm

**Affiliations:** 1 Center for Interdisciplinary Addiction Research (ZIS), Department of Psychiatry and Psychotherapy, University Medical Center Hamburg-Eppendorf (UKE), Martinistraße 52, 20246 Hamburg, Germany; 2 Department of Psychiatry, Medical Faculty, University of Leipzig, Semmelweisstraße 10, 04103 Leipzig, Germany; 3 Laboratory of Demographic Data, Max Planck Institute for Demographic Research, Konrad-Zuse-Str. 1, 18057 Rostock, Germany; 4 Demographic Research Centre, Faculty of Social Sciences, Vytautas Magnus University, Jonavos Str. 66, 44191 Kaunas, Lithuania; 5 Institute for Mental Health Policy Research, Centre for Addiction and Mental Health, 33 Ursula Franklin Street, Toronto, ON M5S 2S1 Canada; 6 Dalla Lana School of Public Health, University of Toronto, 155 College Street, Toronto, ON M5T 3M7 Canada; 7 Department of Health Management, Faculty of Public Health, Lithuanian University of Health Sciences, Tilžės 18, 47181 Kaunas, Lithuania; 8 Health Research Institute, Faculty of Public Health, Lithuanian University of Health Sciences, Tilžės 18, 47181 Kaunas, Lithuania; 9 Department of Preventive Medicine, Faculty of Public Health, Lithuanian University of Health Sciences, Tilžės 18, 47181 Kaunas, Lithuania; 10 Department of Environmental and Occupational Medicine, Faculty of Public Health, Lithuanian University of Health Sciences, Tilžės 18, 47181 Kaunas, Lithuania; 11 Institute of Cardiology, Lithuanian University of Health Sciences, Sukilėlių Av. 15, 50162 Kaunas, Lithuania; 12 Department of Psychiatry, University of Toronto, 155 College Street, Toronto, ON M5T 1P8 Canada; 13 Campbell Family Mental Health Research Institute, Centre for Addiction and Mental Health, 33 Ursula Franklin Street, Toronto, ON M5T 2S1 Canada; 14 Department of International Health Projects, Institute for Leadership and Health Management, I.M. Sechenov First Moscow State Medical University, Trubetskaya Str., 8, B. 2, 119992 Moscow, Russian Federation

**Keywords:** Mortality inequality, Health inequality, Alcohol control policy, Alcohol taxation, Lithuania

## Abstract

**Background:**

Taxation increases which reduce the affordability of alcohol are expected to reduce mortality inequalities. A recent taxation increase in Lithuania offers the unique possibility to test this hypothesis.

**Methods:**

Census-linked mortality data between 2011 and 2019 were used to calculate monthly sex- and education-stratified age-standardized mortality rates for the population aged 40 to 70 years. As primary outcome, we analysed the difference in age-standardized all-cause mortality rates between the population of lowest versus highest educational achievement. The impact of the 2017 taxation increase was evaluated using interrupted time series analyses. To identify whether changes in alcohol use can explain the observed effects on all-cause mortality, the education-based mortality differences were then decomposed into *n* = 16 cause-of-death groupings.

**Results:**

Between 2012 and 2019, education-based all-cause mortality inequalities in Lithuania declined by 18% among men and by 14% among women. Following the alcohol taxation increase, we found a pronounced yet temporary reduction of mortality inequalities among Lithuanian men (− 13%). Subsequent decomposition analyses suggest that the reduction in mortality inequalities between lower and higher educated men was mainly driven by narrowing mortality differences in injuries and infectious diseases.

**Conclusions:**

A marked increase in alcohol excise taxation was associated with a decrease in mortality inequalities among Lithuanian men. More pronounced reductions in deaths from injuries and infectious diseases among lower as compared to higher educated groups could be the result of differential changes in alcohol use in these populations.

**Supplementary Information:**

The online version contains supplementary material available at 10.1186/s12916-022-02721-6.

## Background

Health inequalities are defined as the gap in health indicators between different socioeconomic groupings, e.g. based on income, education or occupation. While life expectancy was growing until the onset of the COVID-19 pandemic in the majority of European countries [1], this health gain was not distributed equally in the population. For instance, even in more egalitarian countries like the Nordic countries, life expectancy increased most in groups with higher, and least in groups with lower socioeconomic status [2, 3].

The existence of health inequalities are widely described (for eight European countries, Japan and South Korea, see, e.g. [4]) and the importance of reducing or eliminating health gaps has been internationally acknowledged [5–7]. To achieve these aims, a comprehensive and multi-faceted set of interventions may be required, spanning across the lifespan and covering behavioural as well as social and economic risk factors [8]. However, it is also conceivable that single interventions can already make a difference and thus serve as entry point to minimize health inequalities. For example, raising tobacco retail prices is more effective in reducing smoking rates among lower- as compared to higher-income smokers [9]. Similarly, raising alcohol retail prices via increased taxation or minimum unit pricing are effective means to improve the health of drinkers [10] and these benefits are expected to be stronger among the more deprived population [11].

While such effects were statistically modelled [12, 13], empirical studies on the impact of alcohol pricing policies on health inequalities are rare. Two studies found evidence that minimum prices as well as changes in affordability were linked to alcohol-related deaths in the hypothesized direction among lower but not among higher educated groups [14, 15]. However, a literature search conducted in preparation for this study found no analyses of health inequalities, e.g. mortality differences.

An opportunity to close this research gap has emerged with the Lithuanian government doubling the excise tax rates for beer, wine and intermediate products as well as increasing excise for absolute ethyl alcohol (relevant for spirits) by 23% on March 1, 2017. This measure has not only decreased affordability of alcoholic beverages [16], but has also been linked to lower all-cause mortality rates [17, 18]. The reduction in national mortality rates were most notable among younger adults [19] and were also observed for liver cirrhosis and suicide deaths [20, 21], which had shown the highest rates among the most socioeconomically disadvantaged populations in various high-income countries [22–24].

According to a recent systematic review, alcohol use explains up to 27% of observed mortality inequalities [25] and Lithuanian studies support this claim [26, 27]. While the contribution of this risk factor differs largely across countries [28], the high alcohol consumption levels and attributable disease burden in Lithuania [29–31] indicates the potential of alcohol control policies to reduce health inequalities in this country. This is also reflected by lower educated persons in Lithuania being 4.6 times more likely to die from alcohol-related diseases as compared to higher educated counterparts [27]. Moreover, absolute mortality and health inequalities appear to be generally greater in Lithuania than in many other European Union countries [28, 32]. Lastly, Lithuanian policy makers have responded to the problem of alcohol harm with strong alcohol control policies, primarily in 2008–2009 and 2016–2018, creating apt conditions to evaluate interventions recommended by the World Health Organization to reduce alcohol harm in a high-income EU country [16, 33].

In this contribution, we seek to evaluate the impact of the major alcohol taxation increase on health inequalities in Lithuania which came into effect on 1st of March 2017. Specifically, we hypothesize that an increase of excise tax was linked to a reduced mortality gap between lower and higher educated persons. The analyses will focus on the population aged 40–70, as health inequalities, alcohol consumption and attributable deaths are most pronounced in this group. The analyses were registered and specified in a published study protocol [34].

## Methods

### Target population and study design

The target population represents adult persons aged 40–70 (for age specification, see below) over the observational period from the 1st of March 2011 (the 2011 census moment) until the 1st of December 2019. The study population was restricted to the participants of the 2011 census aged 40–70 at any time during the study period (total *N* = 1,552,507). As the census only included permanent residents at the census moment, persons migrating to Lithuania after the census were excluded. Based on this population, we calculated monthly estimates of education-specific mortality rates, which were used to derive mortality differences as indicator of health inequalities. Using interrupted time series analyses, we examined whether changes in mortality differences were related to the 2017 taxation increase for beer and wine.

### Data source and preparation

We obtained census-linked mortality data from the years 2011 to 2019 from Statistics Lithuania. The data linkage was performed by Statistics Lithuania following all rules of data confidentiality. The supplied file included time-invariant information collected from census participants (sex, date of birth, and highest educational achievement) as well as the date of death and the cause of death for those who died until December 2019, coded according to the International Classification of Diseases, 10th revision (4-digit code).

The individual-level data were aggregated to obtain a time series of monthly mortality rates, stratified by sex, age group and educational achievement (grouped according to the International Standard Classification of Education, see Additional File 1: “2. Classification of highest educational achievement”). To obtain monthly mortality rates, we calculated the number of deaths (numerator) and the person-months (denominator) by sex, age group and educational achievement. In contrast to cohort studies, the person-months were not accumulated over the study period but represented the number of persons which were at risk of dying in each month within each year of the observational period.

The stratification of deaths and person-months by sex and educational achievement was straightforward as this information was fixed at the 2011 census moment and does not change over time. For age groups, however, we were challenged with a changing composition of the population at risk of dying with each month. We used the *age-of-death* format proposed by Mackenbach and colleagues [35], which involved assigning individuals to that age group which represents their current age in a given month of the observational period. For example, a person turning 50 in January 2015 would be assigned the age group 40–49 in the months leading to December 2014 but would be assigned the age group 50–59 in January 2015 and following months. Lastly, persons emigrating from Lithuania were included in the calculations until the month in which they formally left the country.

Trajectories of the sex-age-education-stratified death counts and person-months are illustrated in Additional File 1: Figure S1 and S2. The age distribution of the person-months in July 2015 (midpoint of the time series) was used to derive weights for calculating a time series of sex-education-stratified age-standardized mortality rates (see Additional File 1: Figure S3). The implausibly low mortality rates in the first month (March 2011, see Additional File 1: Figure S4) was excluded from all analyses, resulting in a time series of *n* = 105 months (April 2011 to December 2019).

### Outcome variables

As primary outcome, we calculated the difference in the age-standardized mortality rates between the population of lowest and highest educational achievement, which gives the excess mortality rate experienced in the lower educated population. As a secondary outcome, we calculated the mortality rate ratio between the population of lowest and highest educational achievement.

Both absolute and relative measures are important as they reflect different perspectives of inequality. The absolute measures show the overall public health importance of inequality in terms of the total excess deaths (per 100,000) related to inequality. Relative measures indicate a relative importance of inequality albeit not considering the number of excess deaths. For example, a relative mortality rate ratio of 1.8 times for rare cause of death may be less important (in terms of public health impact) than the corresponding rate ratio of 1.2 times for a major (frequent) cause of death. Importantly, more pronounced reductions in mortality rates among lower as compared to higher educated groups will result in reductions in absolute inequalities. For reductions in relative inequalities, the percentage change needs to be larger in lower as compared to higher educated groups, which may not always be achieved.

### Intervention variables

To test the effect of the 2017 taxation increase, we considered both level and slope change. The level change was defined as a binary variable, with 0 in all months before (April 2011 to February 2017) and 1 in all months after the intervention (March 2017 to December 2019). The slope change was defined as a continuous variable, with 0 in all months up to (April 2011 to March 2017) and an incremental increase by 1 in all months after the intervention (April 2017 to December 2019; for parametrization of intervention variables, see [36]).

### Confounding variables

In order to rule out alternative explanations, we considered to include time-varying confounders that have been linked to health inequalities in previous studies. Data from seven economic and social variables were available either at a quarterly or annual basis and imputed linearly to obtain monthly time series of each variable (see Additional File 1: Table S1).

### Statistical analyses

As described in the study protocol, we employed generalized additive mixed models to evaluate the intervention impact, separately for primary and secondary outcomes and stratified by sex. The model selection strategy is summarized in the following and explained in greater detail in Additional File 1.

First, the secondary outcome was log-transformed to achieve normality (see Additional File 1: Figures S5 and S6).

Second, baseline models restricted to the pre-intervention period were built following three steps: (1) test for seasonal adjustment, (2) test for time trend, (3) test for confounding variables. For step 3, we examined possible correlations of each outcome variable by sex with the potential confounders (Additional File 1: Table S2). Statistically significant correlations with confounders in the hypothesized direction were only present for absolute mortality difference among men. Based on cross-correlations of confounders and outcome variables (Additional File 1: Figure S5), a 1-month lag of educational expansion was retained as single confounder for the absolute mortality difference among men. For the absolute mortality difference among women, as well as for mortality rates (secondary outcome), no confounders were included in the models (Additional File 1: Table S3).

Third, for the main analyses, three models were built for each outcome by sex, testing (1) an immediate level change, (2) a slope change and (3) a level and slope change. The best performing models were selected based on model fit indicators while taking autocorrelation and stationarity into account (Additional File 1: Table S4 and S5). As no autocorrelation or seasonality was present in the time series (see Additional File 1: Table S3, checked using auto.arima function from R package “forecast” [37]), the analyses were performed using simple generalized linear models (with normally distributed dependent variables) rather than generalized additive models.

The analyses were performed with R version 4.1.2 [38] and all data including the corresponding R code are publicly available (https://doi.org/10.6084/m9.figshare.21749651).

### Additional analyses

In the study protocol, we described to perform additional interrupted time series analyses for alcohol-related mortality rates. However, this was not proven to be feasible given low or even 0 death counts in some months, in particular for the high-educated population. Instead of formal time series analyses on monthly data, we decomposed mortality inequalities into 16 cause-of-death groupings (for definition, see Additional File 1: Table S6) to identify those causes of death that are linked to changes in mortality inequalities.

## Results

### All-cause mortality trends in Lithuania

Between 2012 (first year of complete data) and 2019, all-cause mortality rates declined among lower and higher educated groups aged 40 to 70, resulting in a reduction of absolute mortality differences of (primary outcome, see Table 1). The most pronounced reduction of mortality rates among lower educated men was observed in 2017—the year of the alcohol taxation increase (− 11%). In the same year, mortality also declined strongly in higher educated men, however, not as pronounced as for lower educated men, resulting in a narrowing of mortality differences in that year. Only in 2014, the mortality gap was reduced to a larger degree, following a pronounced reduction of mortality rates among lower but not higher educated men. For women, the absolute and relative reductions in mortality rates among both lower and higher educated groups were less pronounced and there was no indication for the mortality gap to narrow in the year 2017 (+ 2.6%). Across the study period, the absolute mortality gap between lower and higher educated persons fell by 18% for men and by 14% for women.

**Table 1:** Sex-stratified age-standardized all-cause mortality rates (per 100,000) among lower and higher educated persons aged 40–70 in Lithuania and their difference

	Lower educated people		Higher educated people		Difference between lower and higher educated people	
	Mortality rate	Change to previous year (%)	Mortality rate	Change to previous year (%)	Absolute difference in mortality rate^a^	Change to previous year (%)
Men						
2012	1921.0		786.9		1134.2	
2013	1855.4	− 65.7 (− 3.4)	719.4	− 67.4 (− 8.6)	1136.0	+ 1.8 (+ 0.1)
2014	1740.9	− 114.5 (− 6.2)	711.5	− 7.9 (− 1.1)	1029.4	− 106.6 (− 5.8)
2015	1749.1	+ 8.1 (+ 0.5)	743.2	+ 31.6 (+ 4.4)	1005.9	− 23.5 (− 1.3)
2016	1708.2	− 40.9 (− 2.3)	743.7	+ 0.5 (+ 0.1)	964.5	− 41.4 (− 2.4)
2017	1521.5	− 186.7 (− 10.9)	634.7	− 109 (− 14.7)	886.8	− 77.7 (− 4.9)
2018	1504.4	− 17.2 (− 1.1)	637.9	+ 3.2 (+ 0.5)	866.5	− 20.3 (− 1.3)
2019	1506.3	+ 2.0 (+ 0.1)	570.5	− 67.4 (− 10.6)	935.8	+ 69.3 (+ 4.8)
Women						
2012	800.7		315.9		484.7	
2013	799.3	− 1.4 (− 0.2)	313.7	− 2.3 (− 0.7)	485.6	+ 0.9 (+ 0.1)
2014	754.7	− 44.6 (− 5.6)	299.6	− 14.0 (− 4.5)	455.0	− 30.6 (− 3.9)
2015	748.1	− 6.6 (− 0.9)	307.4	+ 7.7 (+ 2.6)	440.7	− 14.3 (− 1.9)
2016	724.1	− 23.9 (− 3.2)	304.1	− 3.2 (− 1.1)	420.0	− 20.7 (− 2.8)
2017	708.1	− 16.1 (− 2.2)	269.9	− 34.2 (− 11.3)	438.2	+ 18.2 (+ 2.6)
2018	668.5	− 39.5 (− 5.6)	264.2	− 5.7 (− 2.1)	404.4	− 33.8 (− 4.8)
2019	684.7	+ 16.1 (+ 2.4)	268.4	+ 4.2 (+ 1.6)	416.3	+ 11.9 (+ 1.8)

^a^rounding errors explain differences between presented mortality rate difference and the single values

### Time series analyses

The interrupted time series analyses suggested a decline in absolute mortality differences (primary outcome, full results see Additional File 1: Table S4) but not for the mortality ratio (secondary outcome, full results see Additional File 1: Table S5) following the alcohol taxation increase implemented in March 2017.

For women, two similar performing models suggested either an immediate (− 3.3 deaths/100,000) reduction or an accumulative decline (− 0.2 deaths/100,000 per month). However, the model fit was overall poor (*R*^2^ = 3.7%) and the results contrasted the increase in annualized mortality differences reported in Table 1. Accordingly, these model findings should be interpreted with caution.

**Fig. 1:**
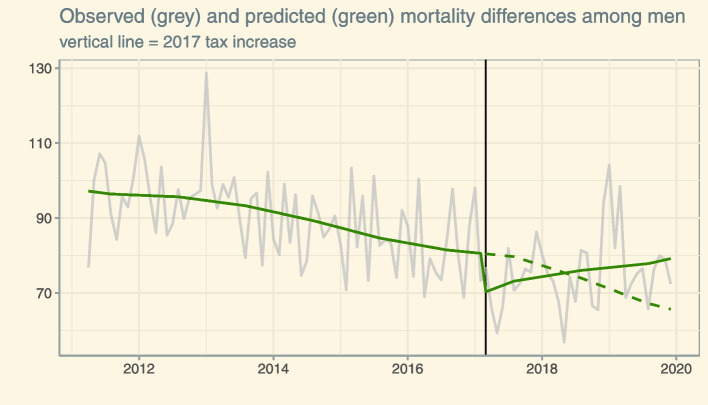
Mortality inequalities among men aged 40 to 70 between April 2011 and December 2019. Green lines are predictions from the interrupted time series model: the green solid line indicates the immediate (reduction) and slope (increase) change related to the March 2017 taxation increase (vertical line), whereas the green dashed line indicates a prediction of mortality inequalities based on the educational expansion only (assuming no policy effects)

For men, the models showed a much better data fit (*R*^2^ = 42%) and the best performing model suggested an immediate reduction of absolute mortality differences equaling to 13% (− 10.3 deaths/100,000; *p* = 0.008) followed by an increasing slope (0.7 deaths/100,000 per month; *p* = 0.0006; see Fig. 1 and Additional File 1: Table S4). Accordingly, the immediate reduction of mortality differences was attenuated in the following months and nullified after 15 months.

Testing the policy effect for mortality ratios did not suggest statistically significant changes in relation to the March 2017 alcohol taxation increase (see Additional File 1: Table S5).

### Cause decomposition

**Fig. 2:**
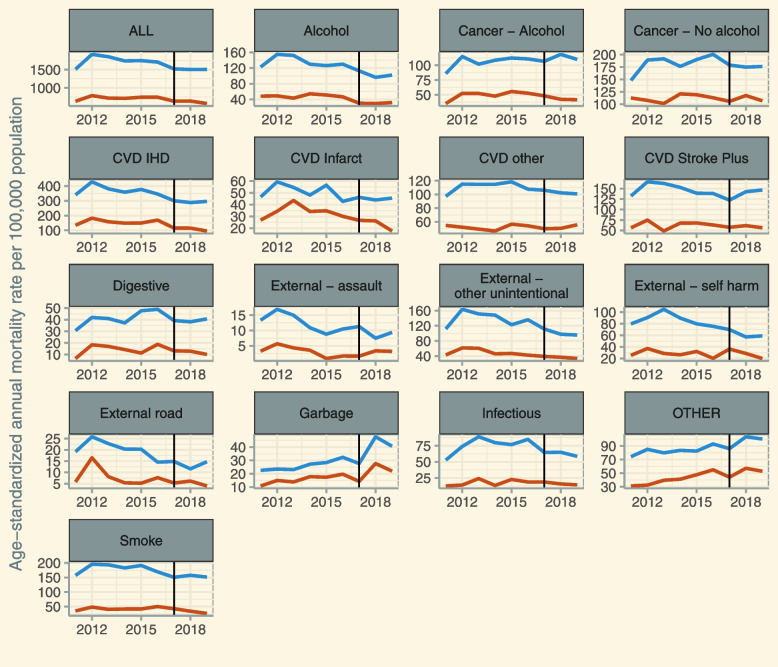
Cause-specific age-standardized mortality rates of lower (blue) and higher (red) educated men aged 40 to 70 in Lithuania, between 2012 and 2019. Vertical line indicates year of alcohol tax increase (2017). For detailed information on cause of death grouping, see Additional File 1: Table S6. Abbreviations: ALL = all-cause mortality; CVD = cardiovascular diseases; CVD IHD = ischemic heart disease; CVD Infarct = Myocardial infarction; Garbage = unknown cause of death; OTHER = causes of deaths not covered in any other category

To further elaborate on the causal pathway of possible reductions in mortality differences linked to the 2017 alcohol taxation increase, we decomposed the annual all-cause mortality differences among men into 16 cause of death groupings. As illustrated in Fig. 2, reductions in mortality rates between 2016 and 2017 among lower educated groups could be visually observed in a number of cause-of-death groupings, such as cancer unrelated to alcohol, stroke, digestive diseases, other unintentional injuries, self-harm, and infectious diseases; except for cancer unrelated to alcohol, all these cause of death groups are causally impacted by alcohol [39]. In all of these cause-of-death groupings, higher educated men did not experience mortality declines to a similar degree, resulting in a narrowing of the respective cause-specific mortality gap in 2017.

**Fig. 3:**
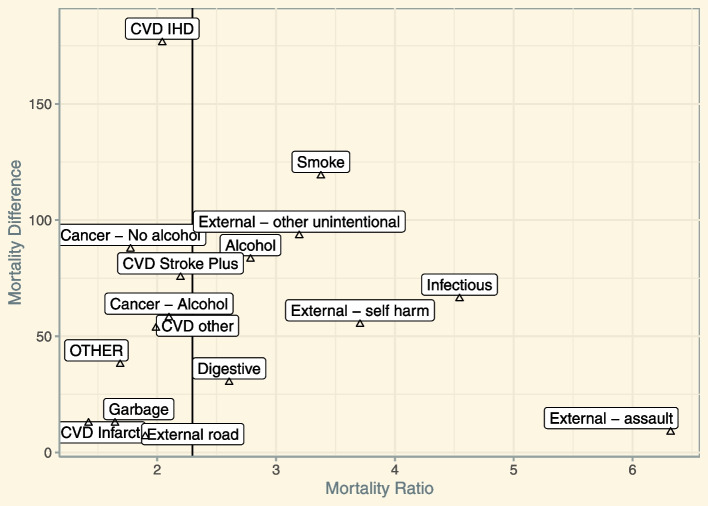
The contribution of different cause-of-death groupings to all-cause mortality differences between lower and higher educated men aged 40 to 70 years old in Lithuania, 2016. The vertical line indicates the all-cause mortality ratio (2.3)

Next, we plotted the mortality ratio against the mortality difference of each of the groups to disentangle their partial contribution to all-cause mortality differences in the year 2016—the reference year prior to the alcohol policy of interest (Fig. 3). First, the mortality differences are all positive, which means that deaths across all cause-of-death groupings occurred more frequently among lower as compared to higher educated men. Second, the educational disparities in all-cause mortality were mainly driven by ischemic heart diseases (18% of all-cause mortality differences), smoking-related diseases (12%), other unintentional injuries (10%), alcohol-unrelated cancer (9%) and alcohol-related diseases (9%). Third, the mortality ratios were similar for most cause-of-death groupings and close to the all-cause mortality gap (rate ratio = 2.3). However, more pronounced rate ratios were observed for infectious diseases (4.5), self-harm (3.7) and assaults (6.3), which made up 13% of the mortality differences in 2016.

Next, we examined how the cause-specific mortality differences have changed between 2016 and 2017 to determine which cause-of-death groupings were driving the 2017 decrease in mortality differences among Lithuanian men. The net reduction of 78 deaths/100,000 (see Table 1) was mainly explained by pronounced reductions in self-harm (28%), other unintentional injuries (27%), and infectious disease (19%; see also Additional File 1: Table S7).

## Discussion

Between 2012 and 2019, education-based all-cause mortality differences in Lithuania declined by 18% among men and by 14% among women aged 40 to 70 years. Following the enactment of a substantial alcohol taxation increase in 2017, we found a pronounced yet temporary reduction of mortality inequalities among Lithuanian men. Subsequent decomposition analyses suggest that the reduction in mortality inequalities between lower and higher educated men was mainly driven by narrowing mortality differences in injuries and infectious diseases.

### Limitations

There are some limitations that should be considered when interpreting our findings. First, interrupted time series analyses are common to evaluate the impact of public policies, but causal inferences from observational study should be drawn with caution. We used mortality differences/ratios as dependent variable, which allowed us to control for secular trends that affected the entire Lithuanian population and to directly estimate the policy effects on a widely used health inequality indicator. An external control group, e.g. lower educated persons from a neighbouring country, would have strengthened our analyses, but such data was not available. In the same line of thinking, the mortality gap may have narrowed because of factors other than changes in alcohol consumption. While alcohol sales point to an abrupt decline of alcohol consumption in 2017 [40], the complete causal pathway leading to a reduction of mortality differences among men could not be assessed in this study.

Another limitation refers to not explicitly modelling the impact of other alcohol policies enacted in Lithuania around the same time, e.g. reduction of sales hours in 2018. However, the 2017 taxation increase was classified as the policy which most likely impacts on health [16] and previous all-cause and cause-specific analyses have also found more support for this than for other policies (e.g. for liver cirrhosis, see [20]). Lastly, we considered to control for several social and economic variables but cannot fully exclude residual confounding. Possibly, weather/temperature, influenza and other external factors may also be related to mortality differences, but as alcohol could potentially play a role in such deaths (e.g. related to freezing or weakening of the immune system), we decided to stick to the list of confounders defined in our study protocol.

### Interpretation of the findings

This study is the first to evaluate whether a substance-related public policy impacts on mortality inequalities. Previous studies have examined the differential effects of smoking bans and alcohol pricing policies on mortality in various socioeconomic groups [14, 15, 41]; however, they did not evaluate the impact on mortality inequalities directly. With our study, we extend the vast literature on using interrupted time series analyses for policy evaluations, e.g. for minimum unit alcohol pricing enacted in Scotland and Wales (UK) [42]. Further, we demonstrate that this tool can be used to evaluate the policy impact on mortality inequalities. The advantage of the employed measure is that it inherently controls for factors that affect the entire population by using less deprived groups as control. The disadvantage is very similar to difference-in-difference analyses, the results of which should be interpreted by taking the trajectory of the control group into account. Accordingly, we did not find a persistent intervention effect on mortality differences among Lithuanian men—despite persistent decreases in all-cause mortality among lower educated groups following the 2017 intervention—because higher educated groups showed strong mortality declines in 2019.

Based on our findings, we conclude that a sufficiently large increase in alcohol excise taxation can contribute to reduce health inequalities. The results suggest, however, that a reduction of mortality differences was not driven by 100% alcohol-attributable diseases but through deaths from infectious diseases and some type of injuries. Why do we find no education-specific impact on 100% alcohol-attributable but on infectious diseases and injuries?

First and foremost, it should be acknowledged that only a minority of alcohol-caused deaths involve 100% alcohol-attributable cause-of-death codings, such as alcoholic liver diseases. Of the nearly 3 million alcohol-attributable deaths estimated globally in 2016, only 5% were coded as alcohol use disorder, while nearly 30% of all alcohol-attributable deaths were estimated to be related to injuries [29]. Thus, considering the wide and detrimental impact of alcohol on health, it is likely to find alcohol policy effects in disease groupings that are not 100% alcohol-attributable.

Moreover, in a previous study, education-specific associations of alcohol affordability and alcohol-specific mortality were found for Finland but not for Sweden [14]. One of the reasons for the non-significant findings with regard to 100% alcohol-attributable deaths in Sweden and for Lithuania in this study could be related to inconsistencies in alcohol death codings. In the abovementioned study, alcohol-specific deaths were defined on both underlying and contributory causes in Finland but not Sweden [14]. Including information on contributory causes may not only increase the validity of diagnoses but also reduce the likelihood of misclassifying alcohol-related deaths [43]. If variance related to coding practices is reduced, the chance of detecting a true intervention effect is increased.

In addition to possible limitations inherent to mortality data, it is conceivable that the education-specific effects of the tax increase enacted in March 2017 were more pronounced for on average moderate rather than heavy drinkers. For people with otherwise low or moderate alcohol intake, a key health risk arises from engaging in episodic heavy drinking occasions, e.g. drinking 6 or more drinks on a weekend. Such pattern is more common among lower educated drinkers [44] and for instance associated with increased injury risks [45]. At the same time, the risk for developing liver cirrhosis at low or moderate drinking amounts and occasional heavy drinking occasions is minimal (for outcome-specific risk curves, see Appendix of [29]). Accordingly, if the price hike resulted in moderate drinkers in the lower educated population to have reduced the number of heavy episodic drinking occasions more so than their higher educated counterparts, this could explain the observed absolute reduction in mortality differences related to injuries. Lastly, reductions in injury and infectious disease mortality are also more likely to occur without any lag time in contrast to possible effects on mortality from chronic diseases such as cancer.

## Conclusions

Using time series analyses, we show that all-cause mortality differences among 40- to 70-year-old Lithuanian men have declined following a large increase in alcohol taxation. The reductions are mainly due to deaths from injuries and infectious diseases, which are also causally impacted by alcohol use and make up a large proportion of alcohol-attributable deaths.

## Supplementary Information

**Additional file 1: Figure S1.** Monthly death count across the study period by sex, age group and education. **Figure S2.** Monthly population count (person-years) across the study period by sex, age group and education. **Figure S3.** Monthly age-standardized mortality rates across the study period by sex and education. **Figure S4.** Monthly death count of lower educated persons for 2011 compared to all remaining years, by sex and age group. **Figure S5.** Cross-correlation of potentially confounding variables with absolute mortality difference among men. **Figure S6.** QQ-plots of dependent variables. **Figure S7.** Time series of the four dependent variables (mortality difference and ratio by sex). **Table S1.** Source and availability of potentially confounding variables. **Table S2.** Correlation of potentially confounding variables with mortality inequalities (dependent variables). **Table S3.** Baseline model selection for time series of *n*=71 months between April 2011 and February 2017. **Table S4.** Main model selection – mortality difference. **Table S5.** Main model selection – mortality ratio (logarithmized). **Table S6.** Cause of death groupings. **Table S7.** Changes in mortality inequalities (absolute difference in age-standardized mortality rate) by cause-of-death grouping for men.

## Data Availability

The aggregated mortality data, i.e. monthly mortality death counts by sex, age, and education and yearly mortality death counts by sex, age, education, and cause-of-death grouping, as well as all covariate data and the R code used to analyse the data are publicly available under https://doi.org/10.6084/m9.figshare.21749651.

## Abbreviations

COVID-19: Coronavirus Disease 2019
EU: European Union
