# Supplementary material for: The impact of alcohol taxation increase on all-cause mortality inequalities in Lithuania: an interrupted time series analysis
Source: BMC Med. 2023 Jan 16;21:22. doi: 10.1186/s12916-022-02721-6 (PMC9841962; doi:10.1186/s12916-022-02721-6)
Supplement: Supplementary file 1 — Additional file 1: Figure S1. Monthly death count across the study period by sex, age group and education. Figure S2. Monthly population count (person-years) across the study period by sex, age group and education. Figure S3. Monthly age-standardized mortality rates across the study period by sex and education. Figure S4. Monthly death count of lower educated persons for 2011 compared to all remaining years, by sex and age group. Figure S5. Cross-correlation of potentially confounding variables with absolute mortality difference among men. Figure S6. QQ-plots of dependent variables. Figure S7. Time series of the four dependent variables (mortality difference and ratio by sex). Table S1. Source and availability of potentially confounding variables. Table S2. Correlation of potentially confounding variables with mortality inequalities (dependent variables). Table S3. Baseline model selection for time series of n=71 months between April 2011 and February 2017. Table S4. Main model selection – mortality difference. Table S5. Main model selection – mortality ratio (logarithmized). Table S6. Cause of death groupings. Table S7. Changes in mortality inequalities (absolute difference in age-standardized mortality rate) by cause-of-death grouping for men. [file 12916_2022_2721_MOESM1_ESM.docx]

Additional File 1

## Manuscript title:

“The impact of alcohol taxation increase on all-cause mortality inequalities in Lithuania: an interrupted time series analysis”

## Authors:

Jakob Manthey, Domantas Jasilionis, Huan Jiang, Olga Meščeriakova, Janina Petkevičienė, Ričardas Radišauskas, Mindaugas Štelemėkas, Jürgen Rehm

***Corresponding author:***

Dr. Jakob Manthey; [j.manthey@uke.de](mailto:j.manthey@uke.de)

Center for Interdisciplinary Addiction Research (ZIS), Department of Psychiatry and Psychotherapy, University Medical Center Hamburg-Eppendorf (UKE), Martinistraße 52, 20246 Hamburg, Germany

# STROBE Checklist

|  | Item No | Recommendation | Page | |
| --- | --- | --- | --- | --- |
| **Title and abstract** | 1 | (*a*) Indicate the study’s design with a commonly used term in the title or the abstract | 1 | |
|  |  | (*b*) Provide in the abstract an informative and balanced summary of what was done and what was found | 3-4 | |
| Introduction | | |  | |
| Background/  rationale | 2 | Explain the scientific background and rationale for the investigation being reported | 5-7 | |
| Objectives | 3 | State specific objectives, including any prespecified hypotheses | 7 | |
| Methods | | |  | |
| Study design | 4 | Present key elements of study design early in the paper | 8 | |
| Setting | 5 | Describe the setting, locations, and relevant dates, including periods of recruitment, exposure, follow-up, and data collection | 8 | |
| Participants | 6 | *Cohort study*—Give the eligibility criteria, and the sources and methods of selection of participants. Describe methods of follow-up | 8 | |
| Variables | 7 | Clearly define all outcomes, exposures, predictors, potential confounders, and effect modifiers. Give diagnostic criteria, if applicable | 8-9 | |
| Data sources/ measurement | 8* | For each variable of interest, give sources of data and details of methods of assessment (measurement). Describe comparability of assessment methods if there is more than one group | 8; Table S1 | |
| Bias | 9 | Describe any efforts to address potential sources of bias | 8-12; Additional file p9,12 | |
| Study size | 10 | Explain how the study size was arrived at | 8 | |
| Quantitative variables | 11 | Explain how quantitative variables were handled in the analyses. If applicable, describe which groupings were chosen and why | 8-10 | |
| Statistical methods | 12 | (*a*) Describe all statistical methods, including those used to control for confounding | 10-12, Additional file p8-15 | |
|  |  | (*b*) Describe any methods used to examine subgroups and interactions | 8-10 | |
|  |  | (*c*) Explain how missing data were addressed | Additional file p8 | |
|  |  | (*d*) *Cohort study*—If applicable, explain how loss to follow-up was addressed | 8 | |
|  |  | (*e*) Describe any sensitivity analyses | 12 | |
| Results | | |  |  |
| Participants | 13* | (a) Report numbers of individuals at each stage of study—eg numbers potentially eligible, examined for eligibility, confirmed eligible, included in the study, completing follow-up, and analysed | 8 |  |
|  |  | (b) Give reasons for non-participation at each stage | N/A |  |
|  |  | (c) Consider use of a flow diagram | N/A |  |
| Descriptive data | 14* | (a) Give characteristics of study participants (eg demographic, clinical, social) and information on exposures and potential confounders | 13; Additional file p8-9 |  |
|  |  | (b) Indicate number of participants with missing data for each variable of interest | N/A |  |
|  |  | (c) *Cohort study*—Summarise follow-up time (eg, average and total amount) | N/A |  |
| Outcome data | 15* | *Cohort study*—Report numbers of outcome events or summary measures over time | Table 1 |  |
| Main results | 16 | (*a*) Give unadjusted estimates and, if applicable, confounder-adjusted estimates and their precision (eg, 95% confidence interval). Make clear which confounders were adjusted for and why they were included | Table 1; Table S4 & S5 |  |
|  |  | (*b*) Report category boundaries when continuous variables were categorized | N/A |  |
|  |  | (*c*) If relevant, consider translating estimates of relative risk into absolute risk for a meaningful time period | Table 1; Table S7 |  |
| Other analyses | 17 | Report other analyses done—eg analyses of subgroups and interactions, and sensitivity analyses | 15-18; Table S7 |  |
| Discussion | | |  |  |
| Key results | 18 | Summarise key results with reference to study objectives | 19 |  |
| Limitations | 19 | Discuss limitations of the study, taking into account sources of potential bias or imprecision. Discuss both direction and magnitude of any potential bias | 19 |  |
| Interpretation | 20 | Give a cautious overall interpretation of results considering objectives, limitations, multiplicity of analyses, results from similar studies, and other relevant evidence | 20-22 |  |
| Generalisability | 21 | Discuss the generalisability (external validity) of the study results | 21-22 |  |
| Other information | | |  |  |
| Funding | 22 | Give the source of funding and the role of the funders for the present study and, if applicable, for the original study on which the present article is based | 24 |  |

# Classification of highest educational achievement

The highest educational achievement documented at census was classified in three categories according to the International Standard Classification of Education. Lower education achievement was defined as “lower than secondary, no education, illiterate or unknown” and involved the following categories:

- vocational school (after graduating from a general upper secondary school); general upper secondary school, gymnasium; vocational school after graduating from a general lower secondary school where, together with a profession; vocational school after graduating from a general lower secondary school where only obtained a profession; vocational school without completion of a general lower secondary school where, together with a profession; vocational (trade) school without completion of a general lower secondary school where only obtained a profession; general lower secondary school; primary school; unfinished primary school; literate (no schooling); illiterate; missing values

Medium educational achievement was defined as “non-vocational or vocational (upper) secondary (completed with a upper secondary diploma)” and involved the following categories:

- professional college; special secondary school (technicum)

Higher educational achievement was defined as “high university or non-university (college) education (completed with a diploma)” and involved the following categories:

- doctoral studies; university (academy, institute, seminary, etc.); college

# Data sources and preparation

The aggregation of the census-linked data set resulted in sex-age-education stratified monthly estimates of the number of deaths and the corresponding person-months. The raw counts are illustrated in **Figure S1 and S2**.


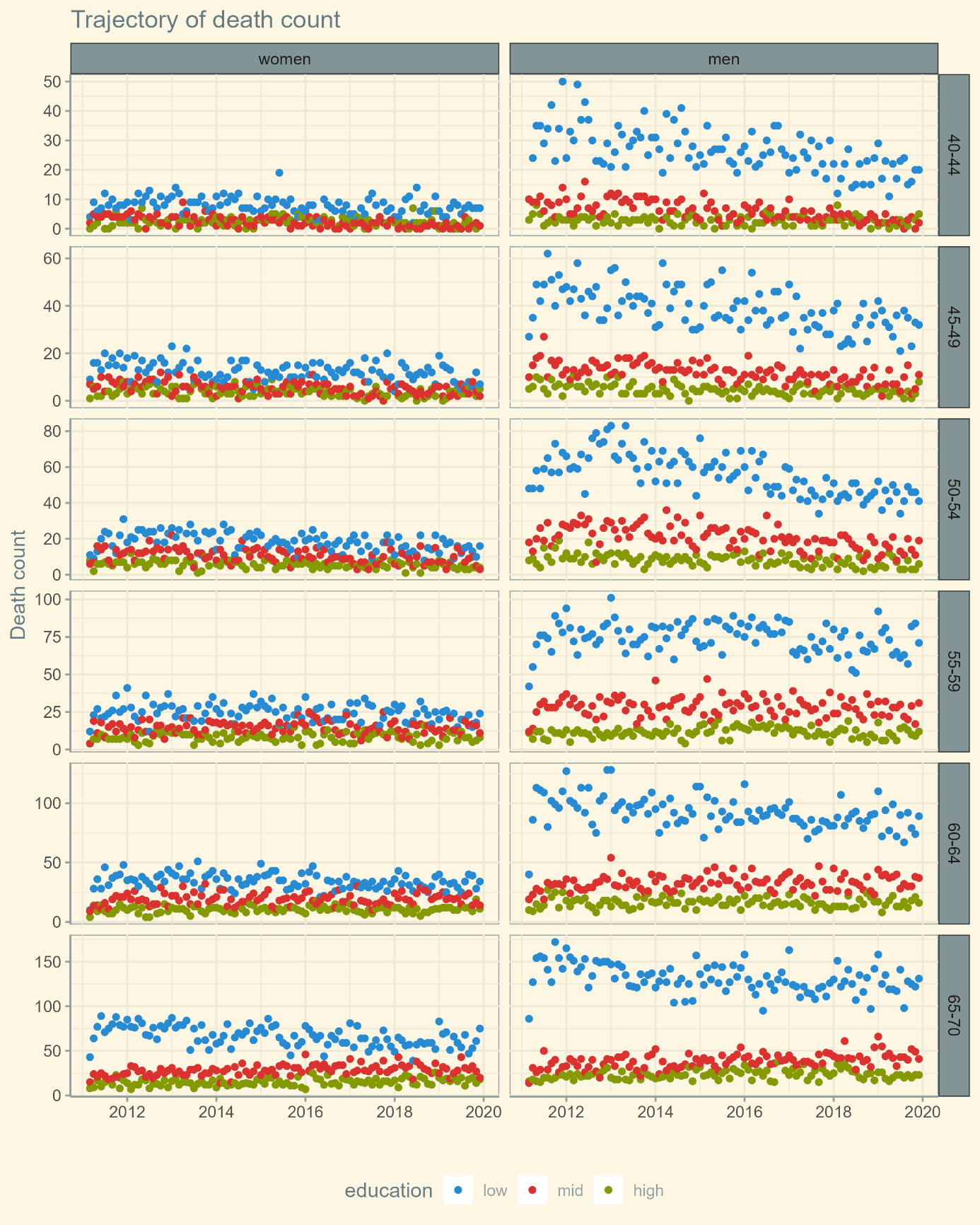


Figure S1. Monthly death count across the study period by sex, age group and education


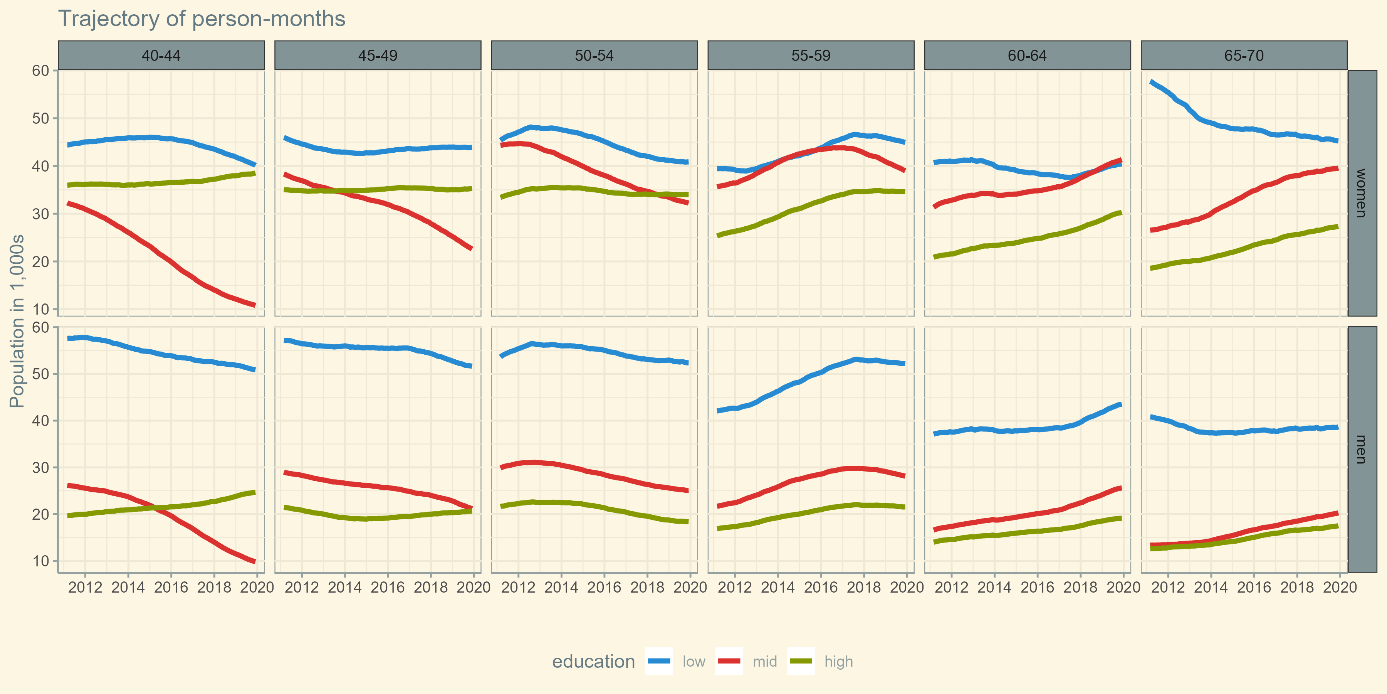


Figure S2. Monthly population count (person-years) across the study period by sex, age group and education


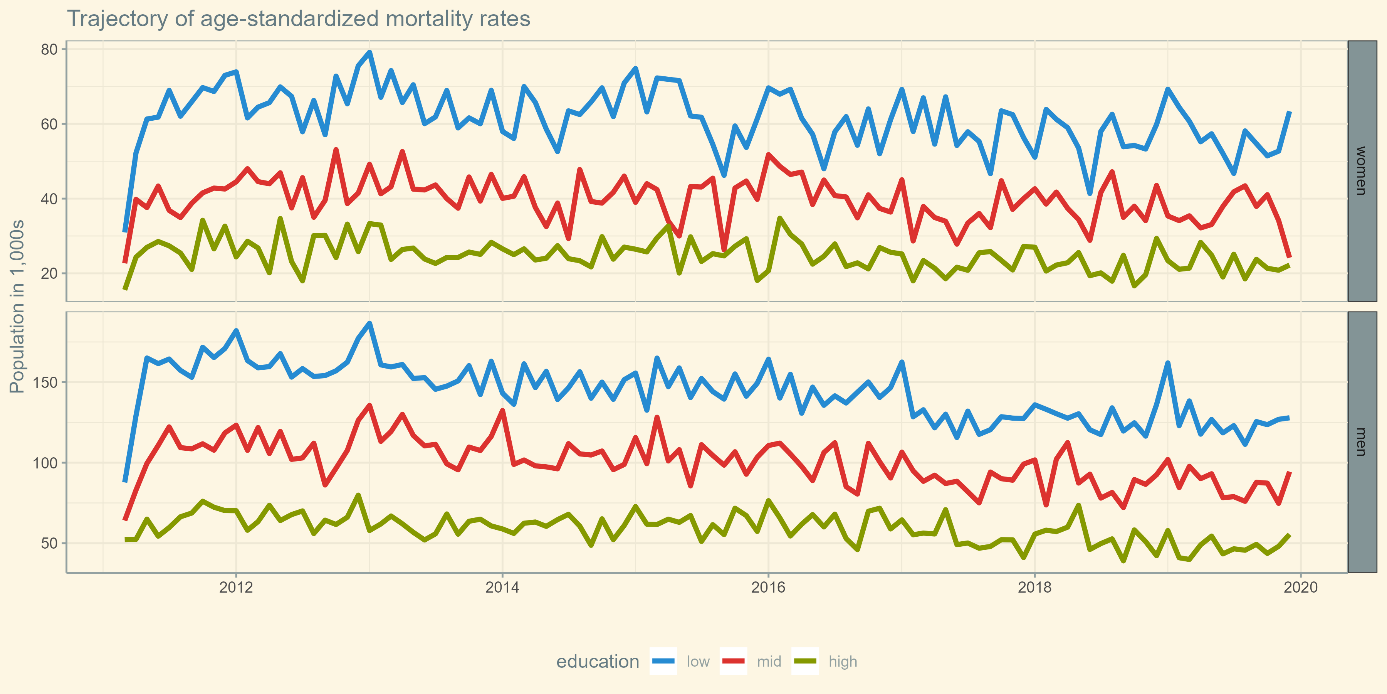


Figure S3. Monthly age-standardized mortality rates across the study perdiod by sex and education

There was an unexpected low number of deaths among lower educated persons in March 2011, resulting in very low mortality rates in this month. As illustrated in **Figure S4**, this was true for both sexes and mainly occurred in the three oldest age groups. Treating this month as outlier, we restricted the analyses to the 105 months April 2011 to December 2019.


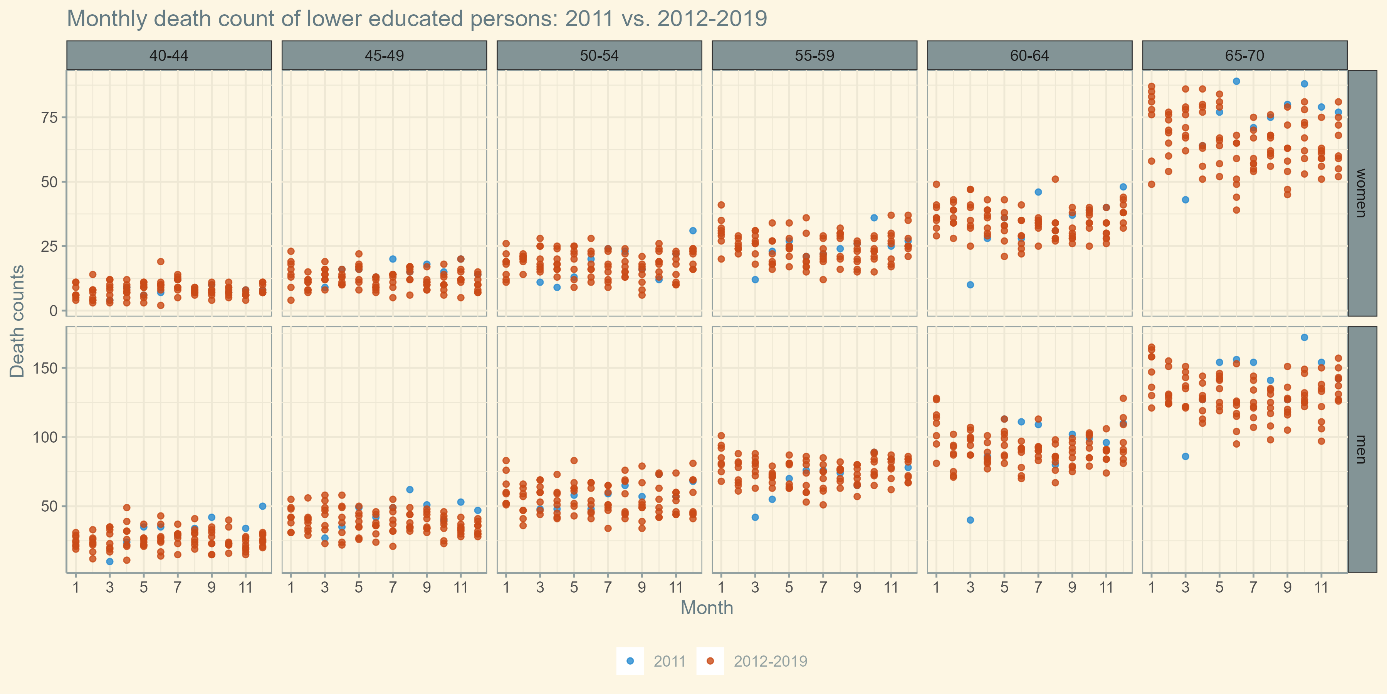


Figure S4. Monthly death count of lower educated persons for 2011 compared to all remaining years, by sex and age group

# Statistical analyses

## Preparation of control variables

As detailed in the study protocol, a number of control variables were considered for inclusion in the time series analyses, including a secular trend in addition to several economic or social indicators (see **Table S1**). The economic and social indicator variables varied by the degree of sociodemographic stratification and temporal availability. Overall, the variable with highest granularity was unemployment rates, which were available on a quarterly basis by sex, age, and educational achievement.

For educational expansion (35-69), poverty risk (18-64) and the ratio of disposable income (15-64), only one age group each was retained given sufficient overlap to the target population. The data concerning unemployment rates were only nearly complete for the age group 20-64, which overlapped satisfactorily with our target group and thus was retained as single age group. For this age group, missing values for women from the year 2016 onwards were recalculated based on estimates from men and the total population.

| **Table S1.** Source and availability of potentially confounding variables | | | |
| --- | --- | --- | --- |
| Variable | By sex, age, education? | Temporal availability | Source |
| Gross Domestic Product | No | Quarterly | Statistics Lithuania |
| Health expenditure | No | Annually | Eurostat: TPS00207 |
| Gini coefficient | No | Annually | Eurostat: ILC_DI12 |
| Ratio of disposable income of highest to lowest quintile | By sex and age | Annually | Eurostat: ILC_DI11 |
| Share of population with high education = educational expansion) | By sex and age | Annually | Eurostat: EDAT_LFS_9903 |
| Poverty risk | By sex and age and education | Annually | Eurostat: ILC_LI07 |
| Unemployment rate | By sex and age and education | Quarterly | Eurostat: LFSQ_URGAED |

## Imputation of control variables

To represent monthly patterns, all variables were linearly imputed. For this purpose, data with annual availability were fixed for July each year and data with quarterly availability were fixed for the middle month in each quarter. Data from 2010 to 2019 were available for all variables, allowing to impute all months from April 2011 to December 2019. For health expenditure, no data from 2020 was available, thus, the last five months (August to December 2019) were forecasted based on carrying on the trend of the preceding year (July 2018 to July 2019).

## Selection of control variables

The potentially confounding variables were next examined in terms of their correlation with each other and with the four outcomes of interest (ratio and difference of mortality rates by sex) in the pre-intervention period. For each of the four outcomes, we obtained a correlation matrix and identified those variables that showed at least a weak correlation with the outcome (spearman r>.35; see **Table S2**). Of those variables, we selected only those correlating in the hypothetical direction. For example, the increase in economic inequality, measured as a rise in the GINI indicator, correlated with a decrease in the mortality gap among Lithuanian men. As this correlation was probably confounded by another variable/process, we did not consider this variable in further analyses.

| **Table S2.** Correlation of potentially confounding variables with mortality inequalities (dependent variables) | | | | |
| --- | --- | --- | --- | --- |
|  | Women | | Men | |
| Variable | Difference | Ratio | Difference | Ratio |
| Gross Domestic Product | -0.23 | -0.03 | -0.47 | -0.21 |
| Health expenditure | -0.16 | -0.01 | -0.47 | -0.26 |
| Gini coefficient | -0.21 | -0.06 | -0.45 ^a^ | -0.23 |
| Ratio of disposable income of highest to lowest quintile | -0.24 | -0.11 | -0.44 ^a^ | -0.21 |
| Share of population with high education (= educational expansion) | -0.16 | -0.01 | -0.47 | -0.26 |
| Poverty risk – low educated | -0.09 | 0.00 | -0.15 | -0.14 |
| Poverty risk – high educated | 0.04 | 0.01 | -0.14 | -0.10 |
| Unemployment rate – low educated | 0.11 | -0.02 | 0.46 | 0.20 |
| Unemployment rate – high educated | 0.16 | 0.04 | 0.45 ^a^ | 0.23 |
| Note. Presented are Pearson correlation coefficients between the four dependent variables and nine potentially confounding variables.  Figures in **bold** are indicative of an at least weak relationship (r>.30) which is in the hypothesized direction.  ^a^ Correlation of sufficient strength but not in the hypothesized direction. | | | | |

Overall, only the absolute mortality differences among men were found to be correlated with any potential confounding variable in the hypothesized direction, namely: gross domestic product, health expenditure, the educational expansion, and the unemployment rates among low educated men. Importantly, these four variables highly correlated with each other (r>.8). In order to avoid introducing multicollinearity in the model, we aimed to choose the variable, which had the best properties as confounder for observed health inequalities. Prior to do testing the variables in statistical models, we examined cross-correlations of the variables to make sure we use the strongest signal. As illustrated in **Figure S5**, the three variables health expenditure, educational expansion and unemployment rate among lower educated persons showed slightly stronger correlations at a lag of one month (k=-1). In other words, they were more predictive of mortality inequalities among men at the next month than at the current month, which would be in line with their hypothesized confounding nature. For gross domestic product, however, we observed the opposite, a stronger signal two months ahead (k=+2). As this is indicative of reverse confounding (mortality inequalities predicting changes in gross domestic product rather than vice versa), we did not consider gross domestic product as confounding variable. Accordingly, only health expenditure, educational expansion and unemployment rates among lower educated persons were considered as control variables in the next analytical steps.


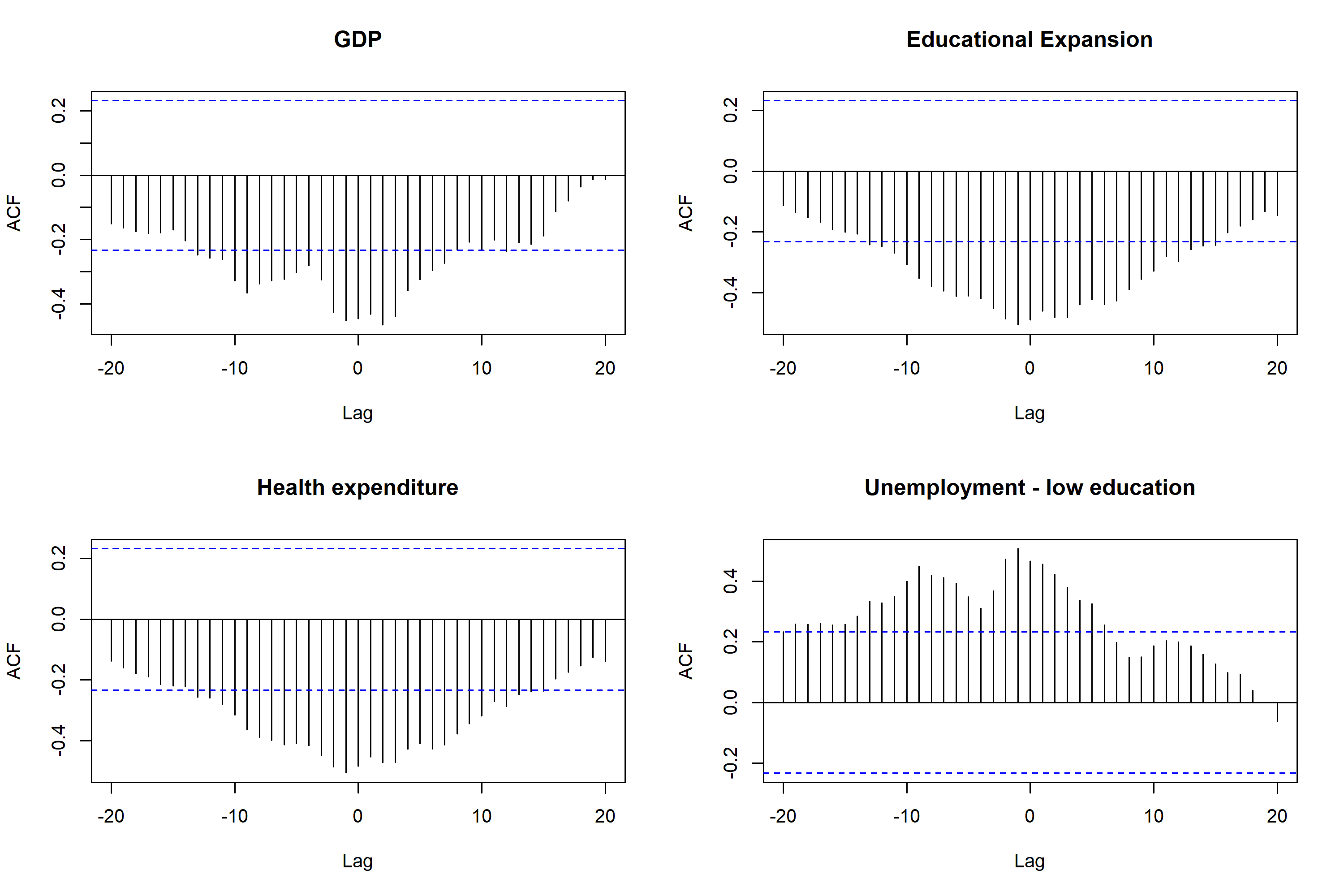


Figure S5. Cross-correlation of potentially confounding variables with absolute mortality difference among men

## Check for normality in dependent variables

Prior to model building, we checked whether each of the four dependent variables was normally distributed. Shapiro-Wilk tests confirmed presence of normal distribution for the mortality difference outcomes for both sexes (women: p=.329; men: p=.07152), but not for the mortality ratios (women: p=.02278; men: p=.01634). Repeating the same tests for logarithmized mortality ratios indicated normally distributed variables (women: p=.1427; men: p=.3313). The distributions of the four variables are illustrated using QQ-plots in **Figure S6**. For further statistical analyses, we used the untransformed mortality differences and the logarithmized mortality ratios (time series are illustrated in **Figure S7**).


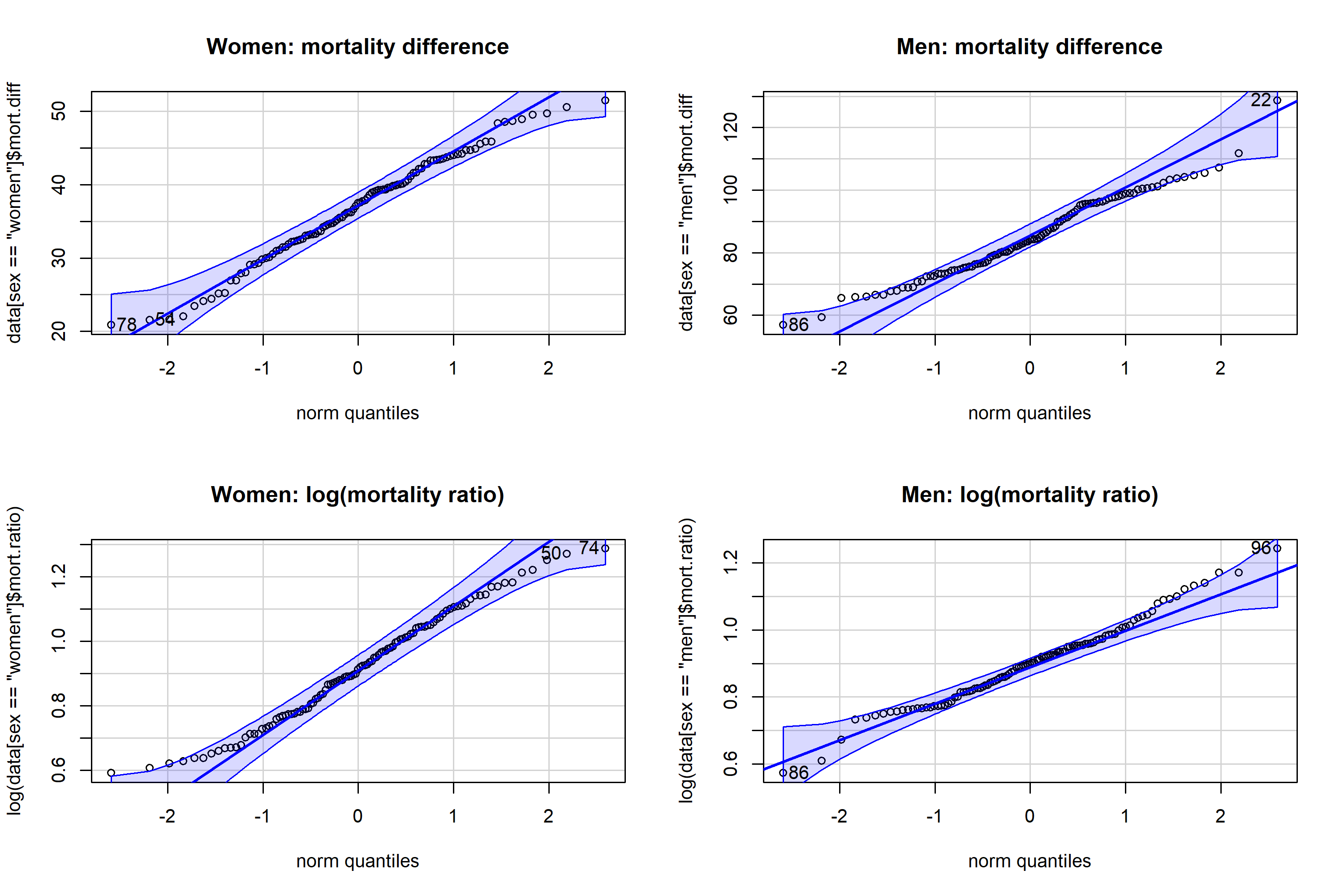


Figure S6. QQ-plots of dependent variables

###
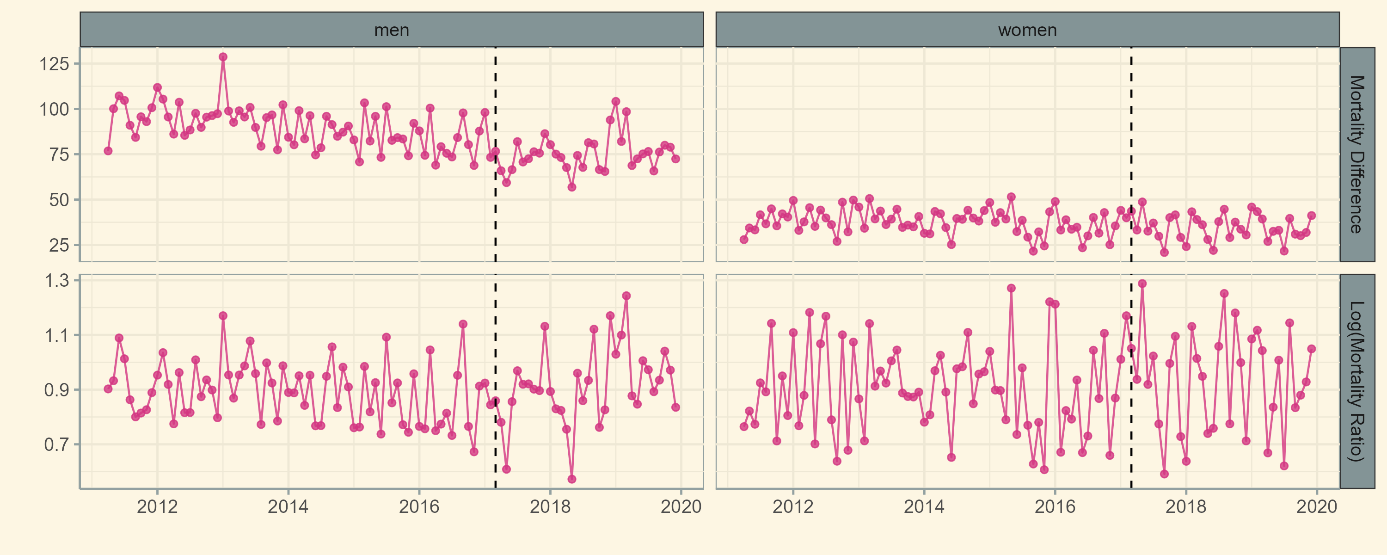


Figure S7. Time series of the four dependent variables (mortality difference and ratio by sex)

## Identifying baseline models

Prior to performing the main analyses, i.e., examining possible intervention effects, we identified baseline models for the first n=71 of months between April 2011 and February 2017, i.e., for all months preceding the intervention, for each dependent variable. Baseline models were chosen from a range of generalized additive mixed models in a sequential manner.

First, we examined the statistical properties of each outcome, i.e., checked for presence of autocorrelation and stationarity. We used the *auto.arima* function (R package “forecast”), examined ACF/pACF plots and performed Ljung-Box tests to identify possible autocorrelation. Stationarity was examined by visual inspection and augmented Dickey-Fuller tests.

Second, we checked whether there are any seasonal patterns in the time series. As detailed in the study protocol, seasonal adjustment was modeled using a monthly spline function in generalized additive mixed models. If the spline function was significant, we checked the properties of the residuals for autocorrelation and stationarity.

Third, we checked whether there was any secular time trend in the data. We only checked for linear time trends because other (e.g., quadratic) trends were not apparent from the plots. As with the second step, the statistical properties were checked in case of significant results.

Fourth, control variables (for selection, see above) were added if they improved model fit. This only concerned the mortality difference among males.

| **Table S3.** Baseline model selection for time series of n=71 months between April 2011 and February 2017 | | | | | |
| --- | --- | --- | --- | --- | --- |
| Sex | Outcome | Variable properties | Step 1: seasonal adjustment | Step 2: linear time trend | Step 3:  other control variables |
| Women | Mortality difference | No autocorrelation; time series is stationary | Not significant (p.=156) | Not significant (p.=15) | Not applicable |
|  | Log(  Mortality ratio) | No autocorrelation according to (p)ACF plots but MA1 term according to *auto.arima*; time series is stationary | Not significant (p.=942) | Not significant (p.=958) | Not applicable |
| Men | Mortality difference | Indications of autocorrelation; stationarity of time series not clear | Not significant (p.=749) | Significant (beta = -0.26; p<.001);  no autocorrelation and residuals are stationary | First lag of each control variable significant (p<.001); no autocorrelation and residuals are stationary in models with health expenditure and educational expansion |
|  | Log(Mortality ratio) | Indications of autocorrelation; time series is stationary | Not significant (p.=818) | Almost significant (beta = -0.001; p=.052);  Indications of autocorrelation but residuals are stationary | Not applicable |

A summary of baseline model selection is given in **Table S3**. For none of the time series, we found a significant seasonal pattern. Non-presence of seasonality made the inclusion of smoothing terms unnecessary. The time series of mortality difference among women showed no signs of autocorrelation and was stationary without further adjustments. The logarithmized mortality ratio among women was similar, however, there were signs of autocorrelation according to the *auto.arima* function, but this was not confirmed by the ACF and pACF plots. Among men, the mortality difference followed a linearly declining trend (monthly reduction of 0.26 deaths per 100,000) in the pre-intervention period. The models with control variables instead of the linear trend showed similar AIC/BIC values, except for the 1-month lag of unemployment rates among lower educated persons. In this model AIC/BIC values were slightly higher and there were signs of 1-year autocorrelation. The best performing model was based on a one month lagged prediction by education expansion. Lastly, for the logarithmized mortality ratio among men, the linear trend was on the edge of becoming significant (p=.052), suggestive of a monthly decline of the mortality ratio in the pre-intervention period by 0.1%.

In summary, the baseline models, describing the four outcomes for the pre-intervention period, were as follows:

- Mortality difference among women: no adjustment required
- Logarithmized mortality ratio among women: no adjustment required
- Mortality difference among men: adjusted by 1-month lag of educational expansion
- Logarithmized mortality ratio among women: adjusted by linear time trend variable

In none of the models, seasonality or for autocorrelation had to be adjusted for. As the outcome variables were also normally distributed, the final analyses were conducted with simple linear regression models.

## Model selection main analyses

Using the baseline models described above, we tested the impact of the alcohol tax increase in March 2017 testing for 1) a level change (sudden increase/decrease of mortality inequalities), 2) a slope change (change in the secular trend of mortality inequalities), and 3) for a level and slope change.

| **Table S4.** Main model selection – mortality difference | | | | | | |
| --- | --- | --- | --- | --- | --- | --- |
|  | WOMEN | | | MEN | | |
| Variable | Model 1 | Model 2 | Model 3 | Model 1 | Model 2 | Model 3 |
| Intercept | 37.907**  (0.833) | 37.702**  (0.786) | 37.907**  (0.835) | 128.875**  (12.270) | 167.601**  (12.664) | 153.304**  (13.525) |
| Level change | -3.237* (1.464) | - | -1.849 (2.506) | -4.539  (3.802) | - | -10.115* (3.933) |
| Slope change | - | -0.158* (0.072) | -0.084 (0.123) | - | 0.507**  (0.190) | 0.715**  (0.202) |
| Educational expansion  (1 month lag) | - | - | - | -1.542**  (0.483) | -3.111**  (0.492) | -2.509**  (0.533) |
| Observations | 105 | 105 | 105 | 105 | 105 | 105 |
| Adjusted R2 | 0.045 | 0.045 | 0.050 | 0.346 | 0.380 | 0.418 |
| Log Likelihood | -353.615 | -353.655 | -353.375 | -395.121 | -392.313 | -388.983 |
| AIC | 711.23 | 711.31 | 712.75 | 796.24 | 790.63 | 785.97 |
| BIC | 719.19 | 719.27 | 723.37 | 806.86 | 801.24 | 799.24 |
| Note. Numbers in brackets are standard errors.  *p<0.05; **p<0.01 | | | | | | |

The candidate models for the mortality difference outcome are summarized in **Table S4**. For women, models with either a level (Model 1) or a slope change (Model 2) were found to be of similar, albeit poor data fit. The model with both level and slope change (Model 3) did not improve model fit as indicated by likelihood ratio tests (p>.1 from Chi² Test). In both Model 1 and 2, a reduction of mortality differences were suggested.

For men, an overall better model fit was found for all three models. Model 3, which included both a level and a slope change variable, showed the best model fit as indicated by lowest AIC and BIC values. Further, likelihood-ratio tests suggested better model fit of Model 3 when compared to the other two models (p<.01 from Chi² Test). According to Model 3, a sudden decrease (-10 deaths per 100,000) followed by an increasing trend (+0.7 deaths per 100,000 per month) of mortality differences was found.

| **Table S5.** Main model selection – mortality ratio (logarithmized) | | | | | | |
| --- | --- | --- | --- | --- | --- | --- |
|  | WOMEN | | | MEN | | |
| Variable | Model 1 | Model 2 | Model 3 | Model 1 | Model 2 | Model 3 |
| Intercept | 0.904**  (0.020) | 0.909**  (0.019) | 0.904**  (0.020) | 0.915**  (0.028) | 0.939**  (0.027) | 0.937**  (0.029) |
| Level change | 0.032  (0.036) |  | 0.047  (0.061) | 0.056  (0.043) |  | -0.012  (0.048) |
| Slope change |  | 0.001  (0.002) | -0.001  (0.003) |  | 0.006**  (0.001) | 0.006**  (0.002) |
| Linear time trend (monthly) |  |  |  | -0.001  (0.001) | -0.001*  (0.001) | -0.001  (0.001) |
| Observations | 105 | 105 | 105 | 105 | 105 | 105 |
| Adjusted R2 | 0.008 | 0.008 | 0.008 | 0.017 | 0.017 | 0.017 |
| Log Likelihood | 36.154 | 35.900 | 36.201 | 73.468 | 77.303 | 77.334 |
| AIC | -68.31 | -67.80 | -66.40 | -140.94 | -148.61 | -146.67 |
| BIC | -60.35 | -59.83 | -55.79 | -130.32 | -138.00 | -133.40 |
| Note. Numbers in brackets are standard errors.  *p<0.05; **p<0.01 | | | | | | |

The candidate models for the (logarithmized) mortality ratio outcome are summarized in **Table S5**. For women, no policy effects were identified in any of these models.

For men, an increasing trend of the mortality ratio following the 2017 taxation increase was suggested in Models 2 and 3, while there were no indications for a sudden decrease of mortality inequalities. A likelihood ratio test indicated no difference between Model 2 and 3 (p>.1 from Chi² test). In the study protocol, we specified to choose the simpler model in case of ambiguity, thus, Model 2 was chosen as best-fitting model.

| **Table S6.** Cause of death groupings | | |
| --- | --- | --- |
| Cause of death group | Description | ICD-10 codes |
| Alcohol |  | F10;G31.2;G62.1;I42.6;  K29.2;K70-K74;K86.0;K85.2;T51;X45;Y15 |
| CVD IHD | Ischemic heart disease but not myocardial infarction | I20;I22-I25 |
| CVD Infarct | Myocardial infarction | I21 |
| CVD Stroke Plus | Stroke + Hypertension + Atrial fibrillation | I60-I69;I10-I15;I48 |
| CVD other | Other CVD codes | I0;I16-I19;I26-I29;I3*;I40-I41;I43-I47; I49;I42;I42.0-I42.5;I42.7;I42.8;I42.9;I5*;I70-I 99;D63.1;E08;E10-E14;N00-N07;N08.0-N08.8;N15.0;N17-N19;Q60-Q62;Q63.0-Q63.2;Q63.8-Q63.9;Q64.2-Q64.9;R73;Z13.1;Z49;Z49.0-Z49.3;Z52.4;Z83.3;Z99.2 |
| Cancer - Alcohol | lip and oral cavity (C00-C08), Nasopharynx and other Pharynx Cancer (C09-C10, C12-C14), Oesophagus cancer (C15), Colon and rectum cancer (C18-C21), Liver cancer (C22), Female Breast Cancer (C50) [Larynx cancer (C32) assigned to smoking] | C00-C08;C09;C10;C12-C14;C15;C18- C19;C20-C22  Women: + C50 |
| Cancer - No alcohol and smoke | All cancer sites not related to smoking and alcohol use | C11;C16;C17;C23-C29;C30-C31;C35-C39;C4;C6-C9;C50-C59;D1-D3];D40-D48;E34.0;K51.4;K62.0-K62.3;K63.5;N60;N84.0;N84.1;N87;Z03.1;Z08;Z09;Z12;Z80;Z85;Z86.0 |
| Digestive | All digestive diseases not as strongly related to alcohol use as liver cirrhosis and pancreatitis (i.e., K70-74; K29.2; K85.2; K86.0) | K20-K23;K25-K28;K29.0-K29.1;K29.3-K29.9;K30- K31;K35-K38;K40-K42;K44-K46;K50;K51.0-K51.3;K51.5-K51.9;K52;K52.2-K52.9;K55-K59;K62.4-K62.6;K62.8-K62.9;K63.0-K63.4;K63.8-K63.9;K67;K68;K75.2;K75.4-K75.9;K76.0-K76.2; K76.4-K76.9;K77.0-K77.8;K80-K83;K85;K85.0-K85.1;K85.3-K85.9;K86.1-K86.9;K87.0-K87.1;K90;K92;K93.8;R11-R19;R85;Z13.81;Z43.1- Z43.4;Z52.6;Z83.7;Z87.1;Z94.4 |
| External - road | Injuries from Road traffic accidents | V0-V89;Y32;Y85 |
| External - other unintentional | Other unintentional injuries and injuries with undetermined intent | V9;W0-W39;W40-W46;W49;W50-W90;X0-X29;X40; X43;X46-X49;X5*;Y10-Y14;Y16-Y31;Y33-Y34;Y40-Y84;Y86;Y88-Y89 |
| External - self harm | Suicide | X60-X79;X80-X84;Y87.0 |
| External - assault | Homicide and other injuries with intentional intent | X85-X89;X9*;Y0*;Y20;Y35-Y36;Y87.1 |
| Infectious | Infectious diseases, partially alcohol-related | A*;B*;J15.9;J15.8;J18;J85.1 |
| Smoke | COPD and cancer sites related to smoking | C32-C34;J40- J44;J47 |
| Garbage | Garbage codes used relatively often in Lithuania | R98;R99 |
| OTHER |  | All remaining codes |

| **Table S7.** Changes in mortality inequalities (absolute difference in age-standardized mortality rate) by cause-of-death grouping for men | | | | | | | | | | | | | | | |  |  |
| --- | --- | --- | --- | --- | --- | --- | --- | --- | --- | --- | --- | --- | --- | --- | --- | --- | --- |
| year | All-cause | Alcohol | CVD IHD | CVD Infarct | CVD Stroke Plus | CVD other | Cancer - Alcohol | Cancer - No alcohol | Digestive | External - road | External - other unintentional | External - self harm | External -assault | Infectious | Smoke | Garbage | OTHER |
| Reference (2016) | 964.5 | 83.2 | 176.4 | 12.7 | 75.4 | 53.5 | 58.0 | 87.6 | 30.2 | 6.9 | 93.4 | 55.2 | 8.8 | 37.2 | 119.0 | 12.7 | 37.9 |
| 2012 | 169.7 | 22.6 | 69.2 | 12.3 | 16.2 | 9.5 | 4.4 | -6.2 | -6.6 | 2.4 | 8.7 | -1.7 | 2.4 | -4.5 | 28.2 | -4.1 | 14.7 |
| 2013 | 171.5 | 25.6 | 46.3 | -1.6 | 38.4 | 11.9 | -8.4 | 2.7 | -6.2 | 7.8 | -2.8 | 21.0 | 1.9 | -5.6 | 33.9 | -3.4 | 2.3 |
| 2014 | 64.9 | -7.9 | 32.8 | 1.2 | 10.0 | 14.7 | 2.8 | -32.1 | -7.2 | 8.1 | 8.7 | 8.2 | -1.5 | 2.0 | 22.3 | -3.3 | 4.4 |
| 2015 | 41.4 | -8.3 | 50.6 | 9.0 | -4.1 | 8.4 | -1.3 | -16.6 | 6.2 | 8.2 | -17.5 | -7.5 | -0.9 | -5.7 | 30.4 | -1.6 | -3.2 |
| 2016 |  |  |  |  |  |  |  |  |  |  |  |  |  |  |  |  |  |
| 2017 | -77.7 | -0.9 | 8.6 | 6.9 | -10.4 | 2.7 | 0.4 | -14.3 | -4.1 | 2.7 | -21.0 | -21.6 | 0.9 | -14.6 | -11.1 | 0.7 | 4.0 |
| 2018 | -98.0 | -16.5 | -3.8 | 5.0 | 5.7 | -1.6 | 18.1 | -30.3 | -4.9 | -1.5 | -32.7 | -26.7 | -4.7 | -12.5 | 4.3 | 7.4 | 8.4 |
| 2019 | -28.7 | -13.3 | 23.7 | 15.2 | 15.0 | -8.4 | 10.3 | -18.3 | 0.4 | 3.8 | -31.5 | -16.2 | -2.6 | -11.7 | 5.7 | 6.0 | 9.4 |
| **Note.** First line indicates the absolute difference in age-standardized mortality rate per 100,000 between lower and higher educated persons in the year 2016. In the subsequent lines, the absolute difference of this measure is reported for various other years (2017 is bold because in this year the intervention of interest was implemented). In the first column, this is reported for all-cause mortality and in the subsequent columns, this is decomposed for various cause-of-death groupings (definition see *Table S6*). | | | | | | | | | | | | | | | | | |
